# Analysis of the Relationship Between Rural-Urban Status and Use of Digital Health Technology Among Older Cancer Survivors Based on the Health Information National Trends Survey: Cross-Sectional Analysis

**DOI:** 10.2196/66636

**Published:** 2025-03-04

**Authors:** Samantha J Werts-Pelter, Zhao Chen, Jennifer W Bea, Amanda E Sokan, Cynthia A Thomson

**Affiliations:** 1 Department of Health Promotion Sciences, Mel and Enid Zuckerman College of Public Health, University of Arizona, 1295 N Martin Ave, Tucson, AZ, 85719, United States, 1 520-869-0340; 2 Department of Epidemiology and Biostatistics, Mel and Enid Zuckerman College of Public Health, University of Arizona, Tucson, AZ, United States; 3 Department of Public Health Practice, Policy, & Translational Research, Mel and Enid Zuckerman College of Public Health, University of Arizona, Tucson, AZ, United States

**Keywords:** cancer, non-metropolitan, disparities, digital divide, health research, aging, rural-urban, digital health technology, cross-sectional, health behaviors, mobile phone

## Abstract

**Background:**

Though telehealth has been a promising avenue for engaging cancer survivors with health care and lifestyle programming, older and rural-dwelling cancer survivors may have additional challenges in accessing digital devices and tools that have not yet been described. This study aimed to use a robust, nationally representative sample collected in 2022 to provide an updated view of digital technology use and the use of technology for health in this population.

**Objective:**

This study aimed to examine the prevalence of digital technology use for health-related activities among older cancer survivors in both rural and urban settings. The primary outcomes of interest included (1) internet access and use for health-related activities, (2) digital device ownership and use as a tool for health behaviors, (3) use of social media for health, and (4) use of telehealth.

**Methods:**

A cross-sectional analysis of the National Cancer Institute’s Health Information National Trends Survey Cycle 6 (HINTS 6) was completed to examine the prevalence of digital technology use among older cancer survivors. For analysis, the sample was restricted to cancer survivors over the age of 60 years (n=710). Unadjusted and adjusted logistic regression models were used to test the association between rurality and digital health tool use.

**Results:**

Overall, 17% (125/710) of the sample lived in a rural area of the United States and the mean sample age was 73 (SD 8.2) years. Older cancer survivors, regardless of rural-urban status, reported a high prevalence of internet usage (n=553, 79.9%), digital device ownership (n=676, 94.9%), and social media use (n=448, 66.6%). In unadjusted models, rural survivors were less likely than urban survivors to report that they had used a health or wellness application in the previous year (odds ratio [OR] 0.56, 95% CI 0.32-0.97; *P*=.04). In adjusted models, rural survivors were more likely to report that they had shared personal health information on social media (OR 2.64, 95% CI 1.13-6.19; *P*=.03). There were no differences in the proportion of rural and urban respondents who reported receiving health services through telehealth in the previous year.

**Conclusions:**

Regardless of the residential status, older cancer survivors report high internet and technology use for health-related activities. These results show promise for the feasibility of using digital technologies to implement supportive care and wellness programming with older cancer survivors.

## Introduction

Improvement in cancer treatments and increasing life expectancy have led to a greater proportion of older, long-term survivors of cancer. As of 2022, 67% of cancer survivors in the United States were above the age of 65 years [1]. Though there are over 12 million older cancer survivors in the United States, their perspectives regarding survivorship and long-term care needs are not well described [1-4]. There are noted cancer care disparities among rural populations where the population tends to be older and to face a higher incidence and mortality rate from cancer than younger survivors. This is likely driven by barriers to accessing state-of-the-art cancer prevention, treatment, and survivorship services that support cancer-preventive behaviors [5-9].

Telehealth and other digital health tools offer an opportunity to bridge the gaps in care between rural and urban cancer survivors. Digital tools, such as the internet, electronic wearable devices, and social media, offer ways to disseminate health promotion materials and education remotely. Given the barriers that rural residents face in accessing in-person health behavior programming and resources, remote delivery with digital health tools is one strategy for delivering this information. In fact, a systematic review of lifestyle behavior change interventions for rural cancer survivors found that programming predominately relied on delivery using a hybrid or remote format by the use of digital technology, though the evidence of effectiveness was limited [10]. Remotely delivered programming to support psychosocial well-being and lifestyle behavior change has been shown to be effective for survivorship more generally, but the evidence of acceptability and usability for survivors living in rural areas is lacking [10]. While digital literacy and access disparities have long existed between rural and urban communities, commonly known as the digital divide [11-13], the COVID-19 pandemic has provided a push to address some of these barriers and has led to greater adoption of telehealth and other digital health tools [14-16]. For example, during the pandemic, providers pushed for the use of telehealth visits to complete routine check-ups with patients, especially immunocompromised cancer patients undergoing treatment. By providing information and instructions on how to complete telehealth visits remotely, many patients became familiar with how to access digital technology. Whether additional modifications in telemedicine and digital health delivery are needed to meet the supportive care needs of older survivors is not known. To address this gap in the literature, additional work is needed to understand the access and use of digital technology in this population.

Digital health technology is a promising tool to support lifestyle behavior change programming, but the acceptability and usability of digital technology for rural cancer survivor populations, particularly older survivors, has not been fully explored. This study aimed to use a robust survey-based dataset to (1) describe the use of digital technology among cancer survivors, (2) assess the use of telehealth and other digital technology to support health, and (3) examine the association between rurality and digital health tool use and access among older (>60 y) cancer survivors who responded to this nationally representative survey in 2022.

## Methods

### Study Design and Population

This cross-sectional analysis was derived from the National Cancer Institute (NCI) Health Information National Trends Survey (HINTS), a nationally representative sample of the adult, noninstitutionalized population in the United States. NCI has been collecting HINTS data every few years since 2003 to evaluate trends in health information access and attitudes toward digital health technology [17,18]. Detailed information about the methodology and publicly available deidentified datasets can be found on the NCI HINTS website [19]. This analysis was deemed exempt by the University of Arizona Institutional Review Board.

This analysis uses data from HINTS 6, which was collected between March and November 2022. As this was the first full HINTS survey completed after the beginning of the COVID-19 pandemic wherein telehealth use increased appreciably [15,20], this iteration was selected to best reflect the current prevalence of digital technology use. HINTS 6 is also the first to include stratification for residential status in the sampling strategy to ensure better representation of rural participants [21]. Out of the 6252 total respondents (response rate 28.1%), those without a history of cancer (n=4982), those under the age of 60 years (n=174), or those with missing data for cancer diagnosis or age (n=119) were excluded from analyses. Cancer diagnosis was determined with a self-report question asking if the respondent had ever been diagnosed with cancer. A cutoff of 60 years of age was used to define an “older cancer survivor” to maximize the analysis sample size and realizing that cancer survivors experience accelerated aging, meaning a 60-year-old cancer survivor may be more like a 65-year-old noncancer survivor. The final analytical sample included 710 cancer survivors 60 years of age or older. Most participants completed HINTS 6 using a mailed paper copy of the survey (435/710, 61.3%) compared with a web-based version of the survey (275/710, 38.7%).

### Ethical Considerations

The HINTS 6 general population survey was designated “exempt research” and approved by the Westat Institutional Review Board (IRB) (Project # 6632.03.51). HINTS 6 was designated as “Not Human Subjects Research” from the National Institutes of Health Office of IRB Operations (iRIS reference number: 562715).

### Outcome Variables

The primary measures of interest pertained to (1) internet access and use for health-related activities, (2) digital device ownership and use as a tool for health behaviors, (3) use of social media for health, and (4) use of telehealth.

#### Internet

Internet use was assessed with the question, “Do you ever go on-line to access the internet or World Wide Web, or to send and receive email?” Respondents who indicated “yes” were then provided a follow-up question asking how they have used the internet for health-related needs in the previous 12 months with 4 statements to consider: “Look for health or medical information,” “Send a message to a health care provider or a health care provider’s office,” “View medical test results,” and “Make an appointment with a health care provider.” Respondents answered each statement with a “yes” or “no” response. Those who reported using the internet were also asked about their level of satisfaction with their home internet connection using a 5-point scale ranging from “extremely satisfied” to “not at all satisfied.”

#### Digital Devices

Survey respondents were asked if they own any of the following digital devices: tablet computer (eg, Apple iPad, Samsung Galaxy, or Kindle Fire), a smartphone (eg, Apple iPhone, Blackberry, or Android), or a basic mobile phone. These responses were then categorized to describe participants who owned these devices, those who owned multiple devices, and those who owned no digital devices. Those who indicated they own a tablet or smart phone were asked, “In the past 12 months, have you used a health or wellness app on your tablet or smartphone?” Ownership and use of wearable devices (eg, Fitbit, Apple Watch, or Garmin Vivofit) was assessed with the question, “In the past 12 months, have you used an electronic wearable device to monitor or track your health or activity?” To assess how respondents have used digital devices to support their health, participants were asked if they had shared any health information from either their wearable device or smartphone with a health professional in the previous year.

#### Social Media

Social media use was assessed with the question, “In the past 12 months, how often did you do the following?” The 5 statements included: “Visited a social media site,” “Shared personal health information on social media,” “Shared general health information on social media,” “Interacted with people who have similar health or medical issues on social media or online forums,” and “Watched a health-related video on a social media site (eg, YouTube).” The response options for each included 5 categorical frequency of use options ranging from “Never” to “Almost every day.” These response types were dichotomized to capture if the respondent had used social media for the purpose at all in the last 12 months or not at all.

#### Telehealth

Telehealth use was assessed with the question, “In the past 12 months, did you receive care from a doctor or health professional using telehealth?” Those who indicated they had not used telehealth were then asked if telehealth had been offered by their provider if they had tried to schedule medical care. Those who did choose to use telehealth in the previous year were prompted with a set of statements and asked to indicate if they agreed that the statement reflected a reason they participated in a telehealth visit.

### Exposure Variables

The exposure of interest for this analysis was residential status. This variable was dichotomized into rural and urban residency using the 2013 Rural-Urban Continuum (RUC) Codes set by the US Department of Agriculture Economic Research Service [22]. This code includes 9-values and is derived along a continuum based on population size and adjacency to a metropolitan area. Based on previous analyses, urban residencies were defined as RUC codes 1‐3, while rural residencies were defined as RUC codes of 4‐9 [11,23,24].

### Sample Characteristics

The sociodemographic characteristics included in this analysis were age, sex, race and ethnicity, occupational status, annual household income, household size, marital status, education, and census region. To ensure adequate cell sample size for analysis, the race and ethnicity variable was condensed into non-Hispanic White, non-Hispanic Black or African American, Hispanic, and non-Hispanic other. Occupational status was organized into 4 categories including employed, retired, disabled, and other (ie, unemployed, homemaker, and student). Related to cancer history, the age of diagnosis, years since diagnosis, and cancer type were included in the summary of the sample.

### Statistical Analysis

This is a cross-sectional analysis of the use of digital health tools and telehealth in older cancer survivors who completed the HINTS 6 survey collected in 2022. Provided survey weights were applied using the Jackknife repeated replication method for population level estimates.

Sociodemographic characteristics, health behaviors, and cancer history were stratified by residential status (rural vs urban) and differences were assessed using Wald design-based chi-square tests of independence or *t* tests. Rurality and associations with using digital health tools and telehealth were individually assessed using both unadjusted and adjusted multiple logistic regression models. Models were adjusted for age, race and ethnicity, annual household income, and education. Sex and marital status were not found to be confounders or effect modifiers and were thus not included in the adjusted model. An α level of .05 was considered statistically significant. A post hoc sensitivity analysis was conducted excluding respondents who reported diagnosis with nonmelanoma skin cancer as this is a population of people with a cancer diagnosis that experience very different treatment and survival outcomes from the general survivor population. All analyses were completed using STATA 17 (StataCorp LLC).

## Results

### Demographics

Out of the 710 older cancer survivors included in this analysis, 17% (n=125) were living in rural areas (see Table 1 for detailed sample characteristics). The average age of the respondents was 73 (SD 8.2) years. While most demographic characteristics did not differ between rural and urban survivors, rural cancer survivors were more likely to identify as non-Hispanic White (93% [n=95] vs 84% [n=415]; *P*=.007) and reported a lower annual household income (*P*=.01). When considering the US census region, a greater percentage of Midwest participants were living in rural areas compared to urban areas (37.3% vs 14.4%), while a lower percentage of participants in the West and Northeast regions were living in rural areas compared to urban areas (*P*<.001). Most respondents were retired (n=497, 71.7%) and married or living with a romantic partner (n=356, 64.7%). Though there were no differences in age at diagnosis or years since diagnosis between groups, nonmelanoma skin cancer was the most prevalent cancer type (n=149, 31.2%); rural participants reported greater prevalence of breast and gynecological cancer than urban residents (*P*=.03). There were no differences in the survey form version completed between rural and urban residents, with 59.9% (n=350) of urban respondents and 68% (n=85) of rural respondents using a paper version of the survey (*P*=.09).

**Table 1.** Sociodemographic characteristics of cancer survivors participating in Health Information National Trends Survey 6 (HINTS 6) by residential status. Missing data <10%.

Characteristics	Total	Rural	Urban	*P* value
Participants, n (%)	710 (100)	125 (17.4)	585 (82.6)	
**Sex, n (weighted %)**				.94
Male Female	316 (46) 393 (54.1)	58 (46.5) 67 (53.5)	258 (45.9) 325 (54.1)	
Age, years, mean (SD)	73.0 (8.2)	72.3 (7.3)	73.2 (8.3)	.29
**Race and ethnicity, n (weighted %)**				.007
Non-Hispanic White Non-Hispanic Black or African American Hispanic Non-Hispanic Other	510 (85.6) 73 (5.7) 47 (5.8) 27 (2.9)	95 (92.8) 7 (2.2) 2 (0.9) 6 (4.2)	415 (84.1) 66 (6.5) 45 (6.7) 21 (2.7)	
**Region, n (weighted %)**				<.001
Northeast Midwest South West	92 (18.6) 114 (18.4) 344 (42.3) 160 (20.7)	12 (9.2) 36 (37.3) 64 (44.9) 13 (8.6)	80 (20.6) 78 (14.4) 280 (41.8) 147 (23.2)	
**Education, n (weighted %)**				.07
Less than high school High school graduate Some college College graduate or more	39 (5) 130 (21.5) 212 (43.1) 324 (30.5)	8 (5.5) 32 (29.4) 43 (44.4) 41 (20.8)	31 (4.9) 98 (19.8) 169 (42.8) 283 (32.5)	
**Occupational status, n (weighted %)**				.28
Employed Retired Disabled Other	138 (20.4) 497 (71.7) 32 (3.3) 35 (4.6)	18 (14.7) 94 (78.9) 6 (1.8) 6 (4.6)	120 (21.6) 403 (70.2) 26 (3.6) 29 (4.6)	
**Annual household income, mean (SD)**				.01
Less than $20,000 $20,000 to <$35,000 $35,000 to <$50,000 $50,000 to <$75,000 $75,000 or more	99 (8.6) 92 (13) 93 (14.8) 128 (23.6) 236 (40)	25 (11.8) 25 (19.1) 13 (13.9) 25 (32) 26 (23.2)	74 (7.9) 67 (11.7) 80 (15) 103 (21.9) 210 (43.4)	
**Marital status, n (weighted %)**				.70
Married or living with a romantic partner Divorced or separated Widowed Single, never married	356 (64.7) 139 (10.4) 152 (15) 59 (9.8)	60 (69.7) 26 (9.7) 28 (15.4) 4 (5.5)	289 (63.8) 113 (10.6) 124 (15) 55 (10.7)	
Age diagnosed, years	57.5 (14.7)	57.5 (14.8)	57.6 (14.7)	.94
**Years since diagnosis, mean (SD)**				.47
<1 2‐5 6‐10 11+	61 (8.3) 137 (20.2) 127 (18.6) 371 (52.9)	11 (9.1) 29 (22.9) 21 (13.1) 62 (55)	50 (8.1) 108 (19.7) 106 (19.7) 309 (52.5)	
**Cancer type, mean (SD)**				.03
Breast Gynecological Colorectal Prostate Blood Skin, nonmelanoma Melanoma Other	99 (16.5) 51 (6.6) 35 (5.4) 78 (14.2) 32 (4.2) 149 (31.2) 36 (8.9) 77 (12.9)	21 (24.7) 14 (12.2) 2 (2.1) 15 (15.9) 3 (2.5) 28 (33.5) 5 (3.9) 7 (5.1)	78 (14.8) 37 (5.4) 33 (6.1) 63 (13.9) 29 (4.5) 121 (30.8) 31 (10) 70 (14.6)	

There were no differences in internet use or mode of access, digital device ownership, or social media use in the previous 12 months between older rural and urban survivors (Table 2). Overall, most older survivors were using the internet (n=553, 79.9%) and predominately used a high-speed service to connect (n=486, 89.8%). Though they shared the same prevalence of internet use, rural cancer survivors were more likely to report a lack of satisfaction with their internet connection than urban survivors (8.7%, n=7, rural vs 0.4%, n=3, urban; *P*<.001). Regardless of residential status, older survivors reported high rate of smartphone ownership (n=544, 78.5%) and only 5% (n=31) reported not owning any digital devices. About half of older survivors owned more than 1 digital device (n=345, 51.7%).

**Table 2.** Weighted prevalence of internet use, digital device ownership, and social media access in the past 12 months by residential status.

	Total, %	Rural, %	Urban, %	*P* value
Used the internet at all	79.9	78.1	80.1	.67
**Mode of accessing the internet** ^**a**^				
Dial-up or telephone line High-speed service Cellular network	1.9 89.8 69.5	2 84.7 74.1	1.9 90.8 68.5	.95 .29 .45
**Internet connection satisfaction**				.006
Extremely satisfied Very satisfied Somewhat satisfied Not very satisfied Not at all satisfied	16.4 47.4 28.8 5.6 1.8	4.6 59.2 22.2 5.3 8.7	18.8 45 30.1 5.7 0.4	
**Digital device ownership** ^**a**^				
58.9 78.5 9.2 51.7 5.1	48.6 76.1 13.6 41.4 3.2	61.1 79 8.2 53.9 5.5	.12 .58 .29 .09 .3
Visited a social media site	66.6	71.6	65.6	.28

a Categories are not mutually exclusive.

### Internet

In both unadjusted and adjusted logistic regression models, the use of the internet to support health did not differ between older rural and older urban cancer survivors. Regardless of residential status, most respondents who used the internet within the past 12 months indicated that they have used the internet to look for health information (n=463, 86.5%), they have sent a message to their health care provider (n=362, 67.5%), and they have viewed their medical test results (n=405, 78.9%; Table 3).

**Table 3.** Association of rural versus urban residence and use of digital health tools in the previous 12 months.

Digital health tools	Weighted percent, %		Unadjusted OR (95% CI)	*P* value	Adjusted OR^a^ (95% CI)	*P* value
	Rural	Urban				
**Internet**						
Used the internet to look for health or medical information	78.7	88.1	0.50 (0.22-1.11)	.09	0.73 (0.33-1.62)	.44
Used the internet to send a message to a health care provider or health care providers office	63.6	68.4	0.81 (0.40-1.61)	.54	0.80 (0.33-1.95)	.62
Used the internet to view medical test results	69.5	80.8	0.54 (0.26-1.11)	.1	0.63 (0.28-1.44)	.27
Used the internet to make an appointment with a health care provider	42.7	58.4	0.53 (0.27-1.07)	.07	0.52 (0.22-1.21)	.13
**Digital devices**						
Used a health or wellness app on a tablet or smartphone	36.9	51.3	0.56 (0.32-0.97)	.04	0.85 (0.47-1.53)	.58
Used an electronic wearable device to monitor or track health or activity	19.8	28	0.64 (0.33-.21)	.17	0.81 (0.38-1.73)	.58
Shared health information from an electronic monitoring device or smartphone with a health professional	21.8	24.4	0.87 (0.46-.63)	.65	1.25 (0.56-2.78)	.58
**Social media**						
Shared personal health information on social media	19	10.9	1.92 (0.82-4.51)	.13	2.64 (1.13- 6.19)	.03
Shared general health-related information on social media (ie, news article)	21.9	26.3	0.79 (0.35-1.79)	.56	0.55 (0.24-1.23)	.14
Interacted with people with similar health or medical issues on social media or online forums	19.7	16.5	1.24 (0.50-3.07)	.63	1.08 (0.46-2.58)	.85
Watched a health-related video on a social media site	40.2	42.3	0.91 (0.48-1.72)	.78	0.76 (0.37-1.55)	.44

a Logistic regression models were adjusted for age, race and ethnicity, annual household income, and level of education.

### Digital Devices

Compared with urban survivors, rural respondents who owned a smartphone or tablet were less likely to report that they had used a health or wellness application in the previous year (36.9%, n=36, rural vs 51.3%, n=234, urban; unadjusted OR 0.56, 95% CI 0.32-0.97; *P*=.04). This difference was no longer significant in adjusted logistic regression models when age, annual household income, education, and race and ethnicity were considered (adjusted OR 0.85, 95% CI 0.47-1.53; *P*=.58). There were no differences in the use of wearable devices to track activity or to share health information with a health care provider. Overall, 26.5% (n=165) of older survivors reported using a wearable device to track their health or activity. While only 23.9% (n=166) of older survivors indicated they have shared data from a smartphone or wearable device with a health professional in the previous year, 81.3% (n=135) indicated that they would be willing to do so in the future.

### Social Media

Two-thirds of older adults (n=448, 66.6%) visited a social media site in the previous year. Though social media use was similar for both urban and rural survivors, older cancer survivors living in rural areas were twice as likely to report that they had shared personal health information on social media in the previous year (adjusted OR 2.64, 95% CI 1.13-6.19; *P*=.03). Less than 20% (n=115) of older cancer survivors reported having used social media or a chat forum to interact with people who have similar health issues and 25.5% (n=173) indicated that they had shared general health information on social media in the previous year.

### Telehealth

There were no differences in the proportion of rural and urban respondents who indicated they had received care from their health care provider using telehealth in the previous 12 months (Table 4). Rural and urban cancer survivors were equally likely to have been offered the option to have a telehealth visit by their providers. For respondents who indicated they received care using telehealth, the primary reason for choosing to participate in a telehealth visit was provider recommendation or requirement (n=215, 81.1%). A total of 54% (n=136) of older cancer survivors indicated that they chose to participate in telehealth because it was more convenient than going to a health care office. Compared with older urban survivors, rural survivors were less likely to indicate that one of their reasons for choosing telehealth was to seek advice about whether in-person care was needed (adjusted OR 0.21, 95% CI 0.05-0.94; *P*=.04).

**Table 4.** Association of rural versus urban residence with use and reasons for use of telehealth in the previous 12 months.

Statement	Weighted percent (%)		Unadjusted OR (95% CI)	*P* value	Adjusted OR^a^ (95% CI)	*P* value
	Rural	Urban				
Received care from a doctor or health professional using telehealth	33.5	42.6	0.68 (0.40-1.15)	.15	0.79 (0.41-1.54)	.49
Offered the option to have a telehealth visit for any medical care	40.6	48.2	0.74 (0.36-1.49)	.39	0.87 (0.41-1.86)	.71
Reported technical problems with the telehealth visits	37.1	26.3	1.65 (0.64-4.24)	.29	1.80 (0.52-6.23)	.35
**Reasons for choosing to participate in telehealth**						
Health care provider recommended or required the visit use telehealth	74.6	82.1	0.64 (0.18-2.32)	.49	0.86 (0.12-6.07)	.87
Wanted advice about whether in-person medical care was needed	10.8	26.4	0.34 (0.12-1.00)	.05	0.21 (0.05-0.94)	.04
Wanted to avoid possible infection at the doctor’s office or hospital	30.4	48.9	0.46 (0.19-1.10)	.08	0.43 (0.13-1.46)	.17
More convenient than going to the doctor	59.9	53.4	1.31 (0.52-3.29)	.56	1.94 (0.63-5.94)	.24
Could include family or other caregivers in the appointment	19.1	23.2	0.78 (0.31-1.98)	.59	0.44 (0.10-1.90)	.26

a Logistic regression models were adjusted for age, race and ethnicity, annual household income, and level of education.

### Sensitivity Analysis

Exclusion of the 149 respondents diagnosed with nonmelanoma skin cancer did not materially change the study results (see Table S1 and S2 in Multimedia Appendix 1 and Multimedia Appendix 2, respectively). In general, no associations were identified between residential status and digital tool use in this smaller sample. There were significant differences in digital device ownership, with rural survivors being less likely to own a tablet computer (37.9%, n=37 vs 56.7%, n=265; *P*=.02) and to own multiple digital devices (28.7%, n=28 vs 51.5%, n=233; *P*=.003; Table S1 in Multimedia Appendix 1).

## Discussion

### Principal Findings

This study is among the first to examine the use of technology among older cancer survivors with a specific focus on the use of technology that became more common overall after the start of the COVID-19 pandemic. While findings from iterations of HINTS completed before the start of the pandemic indicated that rural survivors were less likely to access the internet than urban survivors [25-27], digital health tool access and use were similar between groups in this post–COVID-19 analysis. Regardless of residential status, older survivors of cancer reported a high prevalence of internet use (n=553, 79.9%), digital device ownership (n=676, 94.9%), and social media use (n=448, 66.6%). These post–COVID-19 pandemic prevalence results are similar to those found in an analysis of older cancer survivors who responded to the National Health and Aging Trends Study. In that analysis, a rise in digital health technology use was seen after the pandemic (52% in 2021), compared with before (45% in 2019), though they did not compare rural and urban populations [28]. As this is the first analysis to compare digital technology use between rural and urban older survivors after the pandemic, our results indicate that the pandemic may have enhanced uptake of digital technology across residential areas, potentially reducing the digital divide between rural and urban survivors.

### Comparison With Previous Work

One of the differences noted between groups in this sample was that rural survivors, as compared with urban survivors, were more likely to report that they had shared personal health information on social media in the previous year. There are several hypotheses for why this difference may arise. First, rural survivors experience greater barriers to accessing in-person social support, such as travel distance and access to transportation [5,29,30], which may leave phone or computer-based support as the more feasible option. Second, rural survivors tend to experience greater symptoms of anxiety and depression and poorer health-related quality of life than urban survivors, yet have limited access to mental health professionals [5,8,31,32]. Sharing on social media may be an avenue to garner support, elicit shared experience, or share positive outcomes. There is limited information available on rural cancer survivorship and social media use. As follow-up information was not collected in the HINTS survey about what type of personal health information was shared or who it was shared with, future research should explore how rural survivors use social media and how it is used as a tool for social support or information gathering.

The access and usability results from this study show promise for remote treatment and care for older cancer survivors, particularly those living in rural areas. However, adoption of wearable devices was modest (n=165, 26.5%) and there remains a lack of evidence exploring the barriers to digital technology adoption for those who do not access these tools [33,34]. Wearable devices are one tool to promote self-monitoring of healthy lifestyle behaviors that may be especially beneficial for rural survivors who have do not have access to in-person health coaching. With only 1 in 4 older cancer survivors using a wearable device, additional work is justified to explore the acceptability of wearable devices and to classify the barriers to use. In addition, while these results show promise for increasing acceptability and usability of telemedicine for health care, there is still a question of whether remote delivery of survivorship care and lifestyle behavior change programming is acceptable, feasible, and efficacious for older, rural cancer survivors [10,35]. A recently completed pilot trial examined this question using implementation of a remotely delivered, evidence-based group exercise program for older cancer survivors living in rural areas. This trial, the tele-EnhanceFitness program, incorporated remotely monitored Zoom (Zoom Video Communications) fitness classes 3 days a week for 16 weeks and found low attrition (5%) and high class attendance rate (87%) [36]. Additional research has identified that rural survivors are interested in the incorporation of remote lifestyle programs to their care, but additional study is needed to assess the efficacy of remote programming and barriers to implementation [10,35,37,38].

### Strengths and Limitations

A strength of this study is the use of the HINTS dataset. HINTS is a nationally representative survey, which includes rigorous probability sampling of the US population. The HINTS 6 sampling strategy also introduced stratification for residential status to ensure better representation of rural participants [21]. In addition, the jackknife weighting strategy used for HINTS data analysis allows for population-level comparisons and estimates. Participants used a paper copy to complete the HINTS 6 survey regardless of rural-urban status, limiting potential bias that may have arisen from digital collection of the survey. As with all cross-sectional studies, a limitation of this analysis is the inability to determine cause and effect relationship. The sample size included for analysis was small given the exclusion of any respondents without a cancer history and relied on self-report, which may be affected by recall or response bias. It also lacked diversity reflecting the larger population of cancer survivors, limiting the ability to stratify analysis by race and ethnicity. To maximize sample size and to report results for the overall survivor population, cancer survivors of nonmelanoma skin cancer were included in analysis. This may have introduced confounding by favorable diagnosis, as survivors of nonmelanoma skin cancer generally do not receive chemotherapy or radiation. Another limitation is that the questions regarding digital health technology were general with limited follow-up about satisfaction with the technology or desire for future use. Finally, all data were self-reported, although bias related to reporting of social media use has not been previously reported.

### Future Directions

While digital technology use was found to be similar between older urban and rural survivors, additional research is needed to explore the barriers and facilitators to digital technology adoption for this population and the acceptability of using digital tools for remote intervention delivery. Quantitative work is warranted to examine patterns of digital technology use over time to determine any trends that have emerged since the COVID-19 pandemic. Future work should aim to expand the generalizability by recruiting a diverse sample that better reflects the overall population of cancer survivors living in rural areas.

### Conclusion

These findings provide valuable insight into the acceptability and usability of digital health technology for older cancer survivors. Regardless of rural-urban status, digital health technology use was found to be high among cancer survivors. This is the first analysis of digital health technology use among rural and urban residents after the start of the pandemic, indicating the digital divide may be narrowing as use and access to technology changes over time. For cancer survivors, these results indicate digital technology is a feasible method for delivering health information. Implementation of digital technology-based survivorship and lifestyle programming shows promise as a feasible solution to overcome barriers to high-quality cancer care for older and rural-dwelling survivors, yet additional work in this area is needed.

## Supplementary material

10.2196/66636 Multimedia Appendix 1 Weighted prevalence of internet use, digital device ownership, and social media access in the past 12 months by residential status, excluding those with nonmelanoma skin cancer.

10.2196/66636 Multimedia Appendix 2 Association of rural versus urban residence and use of digital health tools in the previous 12 months, excluding those with nonmelanoma skin cancer.

## Abbreviations

HINTS: Health Information National Trends Survey
IRB: Institutional Review Board
NCI: National Cancer Institute
OR: odds ratio
RUCC: Rural-Urban Continuum Codes
