# Supplementary material for: Analysis of the Relationship Between Rural-Urban Status and Use of Digital Health Technology Among Older Cancer Survivors Based on the Health Information National Trends Survey: Cross-Sectional Analysis
Source: JMIR Cancer. 2025 Mar 4;11:e66636. doi: 10.2196/66636 (PMC11896560; doi:10.2196/66636)
Supplement: Multimedia Appendix 1 [file cancer-v11-e66636-s001.docx]

**Table S1.** Weighted prevalence of internet use, digital device ownership, and social media access in the past 12 months by residential status, excluding those with non-melanoma, skin cancer.

|  | **Total, %** | **Rural, %** | **Urban, %** | **P-value** |
| --- | --- | --- | --- | --- |
| **Used the internet at all** | 77.1 | 70.7 | 78.4 | 0.22 |
| **Mode of accessing the internet^a^**       Dial-up or telephone line       High-speed service       Cellular network | 2.0  87.4  68.7 | 3.9  77.9  70.3 | 1.8  89.2  68.4 | 0.63  0.18  0.83 |
| **Internet connection satisfaction**  Extremely satisfied  Very satisfied  Somewhat satisfied  Not very satisfied  Not at all satisfied | 18.1  45.3  28.9  5.5  2.1 | 4.0  60.8  17.1  5.4  12.8 | 20.5  42.6  31.0  5.6  0.3 | **0.01** |
| **Digital device ownership^a^**       Tablet computer       Smartphone       Basic cell phone       Multiple devices       None | 56.0  76.6  10.9  47.7  4.1 | 37.9  68.6  18.9  28.7  4.4 | 56.7  78.2  9.5  51.5  4.1 | **0.02**  0.14  0.21  **0.003**  0.90 |
| **Visited a social media site** | 65.3 | 67.8 | 64.8 | 0.64 |

^a^Categories are not mutually exclusive.
