# Supplementary material for: Analysis of the Relationship Between Rural-Urban Status and Use of Digital Health Technology Among Older Cancer Survivors Based on the Health Information National Trends Survey: Cross-Sectional Analysis
Source: JMIR Cancer. 2025 Mar 4;11:e66636. doi: 10.2196/66636 (PMC11896560; doi:10.2196/66636)
Supplement: Multimedia Appendix 2 [file cancer-v11-e66636-s002.docx]

**Table S2.** Association of rural vs. urban residence and use of digital health tools in the previous 12 months, excluding those with non-melanoma, skin cancer.

|  | **Weighted percent** **(%)** | | **Unadjusted OR (95% CI)** | **p** | **Adjusted OR^a^ (95% CI)** | **p** |
| --- | --- | --- | --- | --- | --- | --- |
|  | **Rural** | **Urban** |  |  |  |  |
| ***Internet*** | | | | | | |
| Used the internet to look for health or medical information | 80.3 | 87.3 | 0.59 (0.18, 1.98) | 0.39 | 1.31 (0.37, 4.70) | 0.67 |
| Used the internet to send a message to a health care provider or health care providers office | 69.5 | 67.2 | 1.11 (0.53, 2.32) | 0.78 | 1.36 (0.45, 4.08) | 0.58 |
| Used the internet to view medical test results | 70.4 | 79.4 | 0.62 (0.24, 1.56) | 0.30 | 0.84 (0.29, 2.44) | 0.75 |
| Used the internet to make an appointment with a health care provider | 42.4 | 61.2 | 0.47 (0.21, 1.04) | 0.06 | 0.52 (0.17, 1.57) | 0.24 |
| ***Digital Devices*** | | | | | | |
| Used a health or wellness app on a tablet or smartphone | 32.7 | 48.2 | 0.52 (0.25, 1.08) | 0.08 | 0.86 (0.41, 1.80) | 0.69 |
| Used an electronic wearable device to monitor or track health or activity | 13.2 | 23.9 | 0.48 (0.16, 1.46) | 0.19 | 0.79 (0.21, 2.89) | 0.71 |
| Shared health information from an electronic monitoring device or smartphone with a health professional | 21.7 | 25.3 | 0.82 (0.38, 1.78) | 0.61 | 1.42 (0.51, 3.95) | 0.50 |
| ***Social Media*** | | | | | | |
| Shared personal health information on social media | 11.5 | 11.0 | 1.05 (0.40, 2.74) | 0.91 | 1.42 (0.48, 4.17) | 0.52 |
| Shared general health-related information on social media (i.e., news article) | 21.6 | 27.4 | 0.73 (0.31, 1.71) | 0.46 | 0.46 (0.19, 1.13) | 0.09 |
| Interacted with people with similar health or medical issues on social media or online forums | 19.8 | 16.9 | 1.22 (0.47, 3.19) | 0.68 | 1.09 (0.38, 3.13) | 0.88 |
| Watched a health-related video on a social media site | 36.8 | 41.6 | 0.82 (0.40, 1.67) | 0.58 | 0.79 (0.34, 1.82) | 0.58 |
| ***Telehealth*** | | | | | | |
| Received care from a doctor or health professional using telehealth | 33.0 | 42.2 | 0.67 (0.38, 1.19) | 0.17 | 0.81 (0.38, 1.74) | 0.59 |
| Offered the option to have a telehealth visit for any medical care | 40.8 | 47.5 | 0.76 (0.36, 1.60) | 0.46 | 0.94 (0.45, 1.97) | 0.87 |
| Reported technical problems with the telehealth visit(s) | 35.6 | 26.9 | 1.50 (0.52, 4.32) | 0.44 |  |  |

^a^Logistic regression models were adjusted for age, race/ethnicity, annual household income, and level of education.
